# Prevalence and clinicopathological correlates of MSI/dMMR in colorectal cancer: a prospective multicenter registry study from Russia

**DOI:** 10.3389/fonc.2026.1883562

**Published:** 2026-08-18

**Authors:** Dmitrii Semenov, Sergey Achkasov, Natalya Bodunova, Ashot Avdalyan, Alexey Barinov, Anzhelika Chegodar, Ilya Chernikovskiy, Nikita Chizhikov, Mikhail Danilov, Irina Demidova, Vladislav Dmitriev, Dmitry Erygin, Mikhail Fedyanin, Vsevolod Galkin, Daria Ivanilova, Dmitriy Kanner, Nikolay Karnaukhov, Oleg Karpov, Igor Khatkov, Dina Kolesnikova, Ekaterina Kotova, Olga Maynovskaia, Oleg Penzin, Dmitry Pikunov, Andrey Pulin, Nikita Savelov, Anna Semenova, Vitaly Shatalov, Vitaly Shubin, Ilya Sklyar, Oleg Yudin, Evgeniy Zakurdaev, Ilya Pokataev, Alexey Tsukanov

**Affiliations:** 1 Department of Surgery, Clinic K + 31, Moscow, Russia; 2 Administration, Ryzhikh National Medical Research Centre for Coloproctology, Moscow, Russia; 3 Center for Personalized Medicine, SBIH Moscow Clinical Scientific and Practical Center named after A.S. Loginov of DHM, Moscow, Russia; 4 Pathology Department, Moscow Multidisciplinary Clinical Center "Kommunarka", Moscow, Russia; 5 Molecular Biology Laboratory, Oncological Hospital No. 62, Moscow, Russia; 6 Coloproctology Department, Oncological Hospital No. 62, Moscow, Russia; 7 Botkin Hospital, Moscow, Russia; 8 Department of Colorectal Surgery, SBIH Moscow Clinical Scientific and Practical Center named after A.S. Loginov of DHM, Moscow, Russia; 9 Oncology Center No. 1, S.S. Yudin City Clinical Hospital, Moscow, Russia; 10 Moscow Multidisciplinary Clinical Center "Kommunarka", Moscow, Russia; 11 Blokhin National Medical Research Center of Oncology, Moscow, Russia; 12 Pirogov National Medical and Surgical Center, Moscow, Russia; 13 Oncology Department of Antitumor Drug Therapy, Moscow Multidisciplinary Clinical Center "Kommunarka", Moscow, Russia; 14 Oncological Hospital No. 62, Moscow, Russia; 15 Pathology Department, Center for Personalized Medicine, SBIH Moscow Clinical Scientific and Practical Center named after A.S. Loginov of DHM, Moscow, Russia; 16 SBIH Moscow Clinical Scientific and Practical Center named after A.S. Loginov of DHM, Moscow, Russia; 17 Department of Oncology, Pirogov National Medical and Surgical Center, Moscow, Russia; 18 Department of Pathomorphology and Immunohistochemical Studies, Ryzhikh National Medical Research Centre for Coloproctology, Moscow, Russia; 19 Digital Laboratory, Pirogov National Medical and Surgical Center, Moscow, Russia; 20 Department of Oncoproctology, Ryzhikh National Medical Research Centre for Coloproctology, Moscow, Russia; 21 Immunohistochemistry and Molecular Genetics of Solid Tumors, Oncological Hospital No. 62, Moscow, Russia; 22 Department of Laboratory Genetics, Ryzhikh National Medical Research Centre for Coloproctology, Moscow, Russia; 23 Department of Medical Oncology, Oncology Center No. 1, S.S. Yudin City Clinical Hospital, Moscow, Russia

**Keywords:** microsatellite instability, mismatch repair deficiency, colorectal cancer, universal tumour testing, immunotherapy biomarker, prospective registry, real-world data

## Abstract

**Background:**

Microsatellite instability/mismatch repair deficiency are well-established predictive biomarkers for immune checkpoint inhibitor therapy and are used to identify candidates for Lynch syndrome screening in colorectal cancer. In Russia, population-level data on their prevalence are limited — most published series are small, retrospective, or enriched for metastatic disease.

**Methods:**

We performed a pre-specified cross-sectional analysis within a prospective non-interventional registry that enrolled consecutive, unselected patients with primary colorectal adenocarcinoma at six specialised centres in Moscow between August 2022 and December 2025. Of 2,140 patients registered, 2,101 were included after exclusion of synchronous multiple primary tumours. Mismatch repair status was determined by immunohistochemistry, polymerase chain reaction-based microsatellite analysis, or both, following each centre’s standard diagnostic protocol. Logistic regression was used to identify independent clinicopathological predictors of mismatch repair deficiency.

**Results:**

Mismatch repair deficiency was detected in 220 patients, giving an overall prevalence of 10.5% (95% confidence interval 9.2–11.8%). Prevalence was 23.2% in right-sided tumours, 6.2% in left-sided tumours, and 2.8% in rectal tumours (p<0.001). Among graded tumours, poorly differentiated cases had a rate of 32.4% versus 8.4% in low-grade tumours (p<0.001). Female patients accounted for 70.0% of mismatch repair-deficient cases versus 50.1% of proficient cases (p<0.001). On multivariate analysis, right-sided location (odds ratio 3.67; 95% confidence interval 2.57–5.31), high-grade histology (odds ratio 4.15; 95% confidence interval 2.79–6.13), and female sex (odds ratio for male sex 0.49; 95% confidence interval 0.34–0.69) were independently associated with mismatch repair deficiency, while advanced stage (odds ratio 0.64; 95% confidence interval 0.46–0.89) and older age (odds ratio 0.98 per year; 95% confidence interval 0.97–1.00) showed inverse associations. Despite these associations, a meaningful proportion of mismatch repair-deficient cases occurred outside conventional high-risk clinicopathological groups.

**Conclusion:**

Mismatch repair deficiency prevalence in unselected Russian colorectal cancer patients matches international estimates. Clinicopathological features alone are not sufficient to identify all affected patients. To our knowledge, this is the largest prospective multicentre series from Russia to date. Our data support universal mismatch repair testing rather than selective testing based on clinical or pathological criteria (ClinicalTrials.gov, NCT05495776).

## 1 Introduction

Colorectal cancer is still one of the leading causes of cancer death worldwide. In 2020, more than 1.9 million new colorectal cancer cases were estimated worldwide — a number that has been rising steadily, with variation across age groups and geographic regions that is not yet fully explained (1).

Microsatellite instability (MSI)/mismatch repair deficiency (dMMR) are among the most clinically relevant molecular alterations in colorectal cancer. Both result from loss of DNA mismatch repair function. In unselected colorectal cancer series, approximately 15% of tumours show MSI/dMMR; most are sporadic, whereas a minority are caused by germline pathogenic variants in mismatch repair genes and correspond to Lynch syndrome (2). Testing tumours for microsatellite instability or mismatch repair protein loss detects more Lynch syndrome cases than the Amsterdam II and Bethesda criteria (3–5). Russian investigators have also proposed locally adapted criteria for identifying patients with Lynch syndrome, based on tumour MSI status, age, and family history (6).

Beyond hereditary risk, mismatch repair-deficient tumours carry a high mutational burden and prominent immune infiltration, features associated with sensitivity to immune checkpoint blockade (7, 8). This predictive and hereditary-risk role has led major guidelines to incorporate MMR/MSI testing into the diagnostic work-up of colorectal cancer (9). In practice, testing rates and protocols vary considerably between institutions and countries, and reported frequencies differ between populations (5, 10).

Russian data on this question are limited. The largest single-centre series reported a 15% rate among 514 surgically treated colon cancer patients (11). Russian expert consensus documents have highlighted the need for unified approaches to MSI testing and Lynch syndrome diagnostics in routine oncological practice (12), reflecting the fact that diagnostic algorithms for MSI/dMMR testing are not yet standardised across Russian oncology centres, with individual institutions relying on locally available immunohistochemical or molecular methods. However, there are no population-level data from a large consecutive cohort enrolled at multiple institutions in Russia.

To address this gap, we established a prospective registry at six specialised centres in Moscow — to our knowledge the first large-scale, consecutive, multicentre effort of its kind in Russia. Our aim was to estimate real-world mismatch repair deficiency prevalence and describe its clinicopathological correlates in an unselected Russian colorectal cancer population.

## 2 Materials and methods

This analysis draws on data from a prospective non-interventional multicentre registry (ClinicalTrials.gov, NCT05495776). The registry was designed to assess MSI/dMMR prevalence and Lynch syndrome frequency in colorectal cancer patients; the present report covers only tumour-level MSI/dMMR status and its clinicopathological correlates. Germline testing and survival outcomes fall outside the scope of this publication. This report presents a pre-specified cross-sectional analysis within the registry, conducted in accordance with the STROBE Statement for observational studies.

Enrolment ran consecutively from 1 August 2022 to 31 December 2025. The database was locked on 31 December 2025. No changes were made to standard diagnostic or treatment protocols during the study period. A total of 2,140 patients were registered.

Six institutions contributed patients: the National Medical Research Center of Coloproctology named after A.N. Ryzhikh; Moscow City Oncological Hospital No. 62; SBIH Moscow Clinical Scientific and Practical Center named after A.S. Loginov of DHM; Kommunarka Moscow Multidisciplinary Clinical Center; Oncology Center No. 1 of the S.S. Yudin City Clinical Hospital; and S.P. Botkin Moscow Multidisciplinary Clinical Center. The Pirogov National Medical and Surgical Center and Clinic K + 31 provided study coordination and data management but did not enrol patients.

Patients were eligible if they were aged 18 years or older, had histologically confirmed adenocarcinoma of the colon or rectum, and were registered at the time of primary diagnosis before any antitumour treatment for the current tumour. Those who had already received chemotherapy or radiotherapy for the diagnosed colorectal cancer were excluded. A history of treatment for other malignancies was not an exclusion criterion provided that therapy had been completed at least 12 months before enrolment. No stage-based selection was applied. Patients with synchronous multiple primary colorectal cancer were excluded from the present analysis to maintain a homogeneous cohort for the evaluation of MSI/dMMR associations.

MSI/dMMR status was determined according to each centre’s standard diagnostic algorithm. At most centres, immunohistochemical assessment of MLH1, MSH2, MSH6, and PMS2 protein expression was the primary method; loss of expression was defined as complete absence of nuclear staining in tumour cells with an intact internal positive control. Moscow City Oncological Hospital No. 62 used polymerase chain reaction-based microsatellite marker analysis as the primary approach. Where immunohistochemistry was the first-line method, polymerase chain reaction testing was added in equivocal cases. When both results were available, the molecular result took precedence; otherwise the immunohistochemical result was used. MSI/dMMR status was treated as a binary variable throughout.

Registered variables included age, sex, tumour anatomical location, histological grade (G1–G3), and clinical stage categories cT, cN, cM, and cTNM. For analysis, tumour location was grouped into three categories: right-sided, left-sided, and rectal. Right-sided tumours comprised the cecum, ascending colon, hepatic flexure, and transverse colon; left-sided tumours comprised the splenic flexure, descending colon, sigmoid colon, and rectosigmoid junction, following the convention adopted by the National Comprehensive Cancer Network (NCCN) guidelines; rectal tumours were analysed separately. Precise anatomical sub-localisation within the transverse colon and splenic flexure region could not be reliably extracted from source operative and pathology records, so tumours were classified using the standard discrete anatomical categories recorded by treating clinicians. Histological grade was dichotomised into low grade (G1–G2) and high grade (G3). Stage was analysed both in full detail and as a collapsed variable (I–II versus III–IV). No follow-up data are included.

The primary endpoint was MSI/dMMR prevalence in the overall cohort. Secondary endpoints were associations of MSI/dMMR status with age as a continuous variable, age ≤45 years, sex, tumour location, disease stage, and histological grade. The relationship between age and MSI/dMMR status was examined both categorically, using descriptive age bands (predominantly 5-year intervals, with wider terminal categories for the youngest and oldest patients), and continuously, including a restricted cubic spline model to formally assess non-linearity.

Data were collected prospectively in OpenClinica Community Edition (OpenClinica LLC, USA) using a standardised electronic case report form with built-in validation checks. The anonymised dataset was exported in CSV format for analysis.

All statistical analyses were performed in R version 4.5.2 (R Foundation for Statistical Computing, Vienna, Austria). Continuous variables are summarised as mean ± standard deviation and median with interquartile range; categorical variables as counts and percentages. Group comparisons used the Pearson chi-square test or Fisher’s exact test as appropriate, applied without continuity correction given the large sample size. Logistic regression was used to identify independent predictors of MSI/dMMR status; the model included variables that were clinically relevant or statistically significant on univariate analysis, and multicollinearity was assessed using variance inflation factors (VIF). Model fit was evaluated using the Hosmer-Lemeshow goodness-of-fit test, and discriminative performance was assessed using the area under the receiver operating characteristic curve (AUC). Effect estimates are reported as odds ratios with 95% confidence intervals. Only patients with complete data across all model variables were included in the multivariate analysis; a sensitivity analysis compared patients with known versus missing histological grade across age, sex, tumour location, stage, and MSI/dMMR status. All tests were two-sided; p<0.05 was the threshold for statistical significance.

The protocol was approved by the Independent Ethics Committee of the National Medical Research Center of Coloproctology named after A.N. Ryzhikh (protocol No. 10/22, 23 June 2022). Written informed consent was obtained from all participants. The study was conducted in accordance with the Declaration of Helsinki and Good Clinical Practice.

## 3 Results

All 2,140 enrolled patients had MSI/dMMR status recorded. Thirty-nine were excluded for synchronous multiple primary colorectal cancer — six of them MSI/dMMR-positive — leaving 2,101 patients in the analytical cohort.

Patient numbers by centre: SBIH Moscow Clinical Scientific and Practical Center named after A.S. Loginov of DHM, 521 (24.8%); Kommunarka Moscow Multidisciplinary Clinical Center, 497 (23.7%); Moscow City Oncological Hospital No. 62, 431 (20.5%); National Medical Research Center of Coloproctology named after A.N. Ryzhikh, 392 (18.7%); S.P. Botkin Moscow Multidisciplinary Clinical Center, 143 (6.8%); Oncology Center No. 1 of the S.S. Yudin City Clinical Hospital, 117 (5.6%).

### 3.1 Cohort characteristics

Clinicopathological characteristics stratified by MSI/dMMR status are shown in **Table 1**. Women slightly outnumbered men: 1,096 (52.2%) versus 1,005 (47.8%). Mean age was 66.7 ± 11.5 years; median 68 years (interquartile range 61–75; range 24–95). Patients aged 45 or younger made up 5.5% of the cohort (115/2,101). The 66–70 and 71–75 year groups were the largest, at 19.1% and 18.5% respectively; those aged 18–40 and 41–45 accounted for 2.8% and 2.7%.

**Table 1:** Clinicopathological characteristics of MSS/pMMR and MSI/dMMR patients.

Variable	MSS/pMMR (n=1,881)	MSI/dMMR (n=220)	p-value
Age, years, mean ± SD	66.8 ± 11.3	66.1 ± 13.5	0.518
Age group			<0.001
>45 years	1,789 (95.1%)	197 (89.5%)	
≤45 years	92 (4.9%)	23 (10.5%)	
Sex			<0.001
Female	942 (50.1%)	154 (70.0%)	
Male	939 (49.9%)	66 (30.0%)	
Tumor location			<0.001
Right-sided	481 (25.6%)	145 (65.9%)	
Left-sided	917 (48.8%)	61 (27.7%)	
Rectal	483 (25.7%)	14 (6.4%)	
AJCC stage group			0.021
I-II	746 (39.7%)	105 (47.7%)	
III-IV	1,135 (60.3%)	115 (52.3%)	
Tumor differentiation*			<0.001
G1-G2	1,417 (92.1%)	130 (69.1%)	
G3	121 (7.9%)	58 (30.9%)	

*Percentages for tumor differentiation calculated among patients with available data (n=1,726; 375 missing). Missing grade data were not significantly associated with age (p=0.599), sex (p=0.185), or MSI/dMMR status (10.9% vs. 8.5% among patients with known grade; p=0.176), but were significantly associated with tumour location (p<0.001) and stage (p=0.001), and were concentrated at one centre.

Left-sided colon tumours were most common (978 cases, 46.5%), followed by right-sided (626, 29.8%) and rectal (497, 23.7%).

Stage III was the most frequent presentation (788 patients, 37.5%), then stage II (567, 27.0%), stage IV (462, 22.0%), and stage I (284, 13.5%). Stages III–IV combined accounted for 59.5% of the cohort. Histological grade was not recorded for 375 patients (17.8%); among the 1,726 with available data, G2 was predominant (920, 53.3%), followed by G1 (627, 36.3%) and G3 (179, 10.4%).

### 3.2 MSI/dMMR prevalence

MSI/dMMR was found in 220 of 2,101 patients — a prevalence of 10.5% (95% confidence interval 9.2–11.8%).

Rates differed across centres (χ² = 12.706; p = 0.026), from 7.7% (95% confidence interval 3.9–13.3%) at S.P. Botkin Moscow Multidisciplinary Clinical Center to 13.8% (95% confidence interval 11.0–17.1%) at SBIH Moscow Clinical Scientific and Practical Center named after A.S. Loginov of DHM. No pairwise difference survived Bonferroni correction.

### 3.3 Clinicopathological associations

Tumour location showed the strongest association with MSI/dMMR. Rates were 23.2% (95% confidence interval 20.0–26.7%; 145/626) for right-sided tumours, 6.2% (95% confidence interval 4.8–8.0%; 61/978) for left-sided, and 2.8% (95% confidence interval 1.5–4.7%; 14/497) for rectal (χ² = 157.32; p < 0.001). Within the left-sided group, the splenic flexure subgroup showed a numerically higher rate (17.2%; 15/87) than the remainder of the left colon. This is compatible with the splenic flexure’s position at the embryological watershed between the midgut and hindgut, which may confer a molecular phenotype intermediate between classically right- and left-sided tumours; the subgroup was nonetheless classified as left-sided in the primary analysis, following the convention adopted by the NCCN guidelines.

MSI/dMMR was more common in women: 70.0% of positive cases were female versus 50.1% of MSS/pMMR cases (χ² = 31.322; p < 0.001).

**Figure 1:**
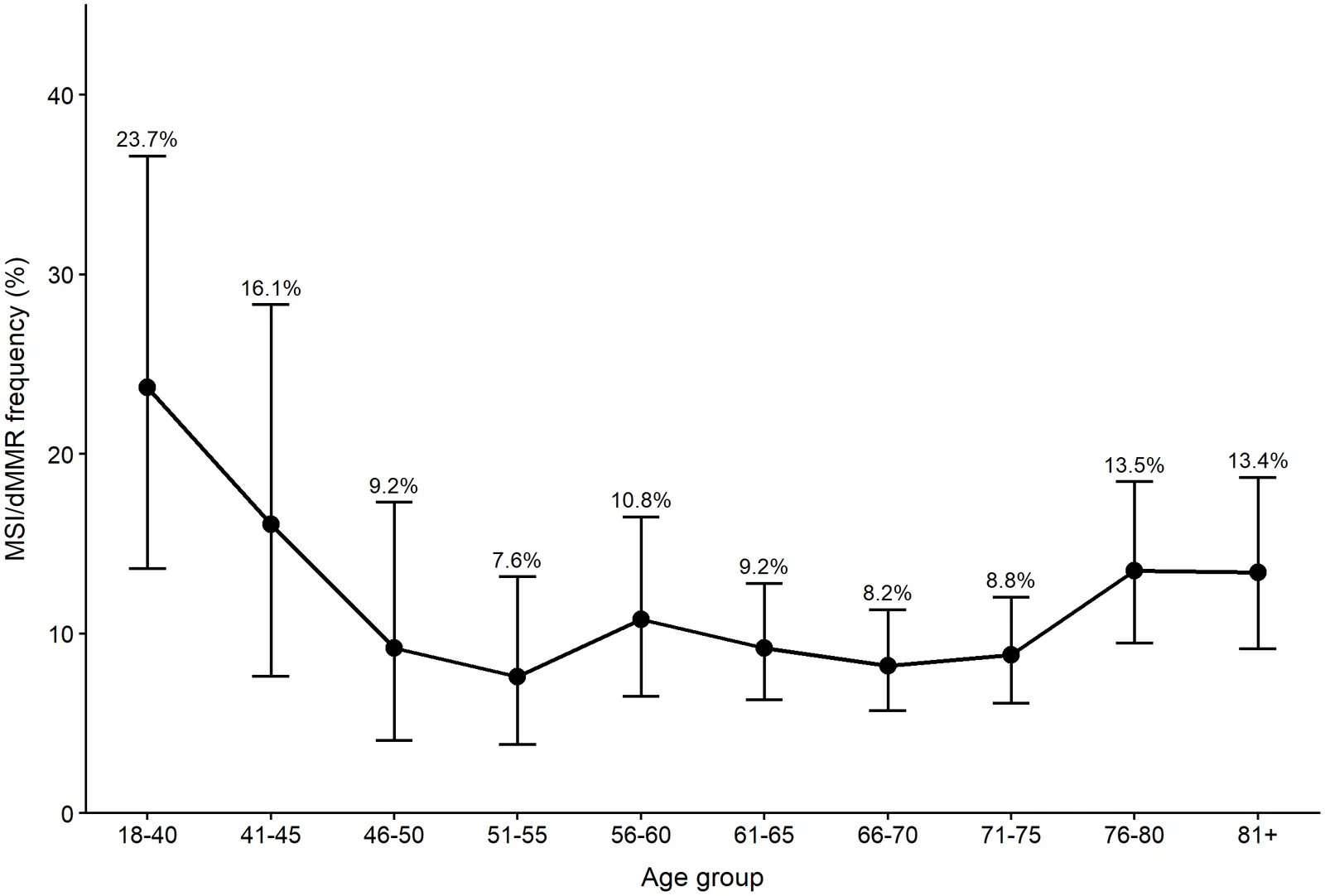
Age-specific frequency of MSI/dMMR in colorectal cancer. Error bars represent 95% confidence intervals.

Patients aged 45 or younger had a higher rate than older patients: 20.0% versus 9.9% (χ² = 11.783; p < 0.001). Age-group analysis showed a non-linear pattern (χ² = 22.813; p = 0.007): highest in the 18–40 group (23.7%), declining through 41–45 (16.1%), 46–50 (9.2%), and 51–55 (7.6%), relatively flat between 56 and 75 years (8.2–10.8%), then rising at 76–80 (13.5%) and 81 and older (13.4%) (**Figure 1**). Mean age was virtually identical between MSI/dMMR-positive and MSS/pMMR patients (66.1 ± 13.5 versus 66.8 ± 11.3 years; p = 0.518). To formally test this non-linearity, age was modelled using restricted cubic splines (3 degrees of freedom) in place of the linear term in the multivariate model described below. The spline model showed a modestly better fit than the linear-age model (likelihood ratio test, p = 0.035; AIC 1013.4 versus 1016.1), lending some support to a non-linear age effect, although the improvement in fit was modest.

Stage-specific analysis showed that MSI/dMMR prevalence was highest at stage II (13.4%; 95% confidence interval 10.7–16.5%), followed by stage III (10.9%; 95% confidence interval 8.8–13.3%), stage I (10.2%; 95% confidence interval 6.9–14.3%), and stage IV (6.3%; 95% confidence interval 4.2–8.9%). Differences across the four stage groups were statistically significant (χ² = 14.056; p = 0.003). The contrast between stage II and stage IV was particularly pronounced (odds ratio 0.43; 95% confidence interval 0.27–0.69; p < 0.001), while the difference between stage I and stage IV did not reach significance (p = 0.066).

Among graded tumours, MSI/dMMR rates were 9.4% (95% confidence interval 7.2–12.0%) in G1, 7.7% (95% confidence interval 6.1–9.6%) in G2, and 32.4% (95% confidence interval 25.6–39.8%) in G3 (χ² = 96.304; p < 0.001). Rates in G1 and G2 were not significantly different from each other (odds ratio 0.81; p = 0.263), while G3 showed markedly higher odds compared to both G1 (odds ratio 4.60; p < 0.001) and G2 (odds ratio 5.72; p < 0.001), confirming that the distinction between low-grade and high-grade tumours captures the relevant biological boundary.

Regression results are in **Table 2**. On univariate analysis, MSI/dMMR was associated with age ≤45 years (odds ratio 2.27; 95% confidence interval 1.38–3.61; p < 0.001), right-sided location (odds ratio 4.53; 95% confidence interval 3.31–6.27; p < 0.001), G3 histology (odds ratio 5.22; 95% confidence interval 3.63–7.48; p < 0.001), stage III–IV (odds ratio 0.72; 95% confidence interval 0.54–0.95; p = 0.021), and male sex (odds ratio 0.43; 95% confidence interval 0.32–0.58; p < 0.001). Age as a continuous variable was not significant on univariate analysis (odds ratio 1.00; 95% confidence interval 0.98–1.01; p = 0.454).

**Table 2:** Univariate and multivariate logistic regression analysis of factors associated with MSI/dMMR.

Variable	Univariate OR (95% CI)	p-value	Multivariate OR (95% CI)	p-value
Age (per year)	1.00 (0.98-1.01)	0.454	0.98 (0.97-1.00)	0.016
Age ≤45 years	2.27 (1.38-3.61)	<0.001	—	—
Right-sided location	4.53 (3.31-6.27)	<0.001	3.67 (2.57-5.31)	<0.001
Rectal location	0.44 (0.23-0.76)	0.006	0.52 (0.27-0.95)	0.042
High grade (G3)	5.22 (3.63-7.48)	<0.001	4.15 (2.79-6.13)	<0.001
Stage III-IV	0.72 (0.54-0.95)	0.021	0.64 (0.46-0.89)	0.007
Male sex	0.43 (0.32-0.58)	<0.001	0.49 (0.34-0.69)	<0.001

Left-sided location is the reference category for tumor location (comprising the splenic flexure, descending colon, sigmoid colon, and rectosigmoid junction, per NCCN convention). Age ≤45 years was not entered into the multivariate model due to collinearity with continuous age.

The multivariate model retained right-sided location (odds ratio 3.67; 95% confidence interval 2.57–5.31; p < 0.001), G3 histology (odds ratio 4.15; 95% confidence interval 2.79–6.13; p < 0.001), stage III–IV (odds ratio 0.64; 95% confidence interval 0.46–0.89; p = 0.007), male sex (odds ratio 0.49; 95% confidence interval 0.34–0.69; p < 0.001), and continuous age (odds ratio 0.98; 95% confidence interval 0.97–1.00; p = 0.016) as independent predictors. The binary age ≤45 variable was excluded due to collinearity with continuous age. Variance inflation factors for all model variables were low (1.01–1.06), indicating no meaningful collinearity. The model showed good calibration (Hosmer-Lemeshow test, p = 0.417) and acceptable discrimination (area under the receiver operating characteristic curve 0.771; 95% confidence interval 0.733–0.809).

### 3.4 MSI/dMMR testing methods

The testing method was documented for all 220 MSI/dMMR-positive patients. Immunohistochemistry alone was used in 107 cases (48.6%); polymerase chain reaction-based methods in 113 (51.4%). Immunohistochemistry was the sole method at Oncology Center No. 1 of the S.S. Yudin City Clinical Hospital and Kommunarka Moscow Multidisciplinary Clinical Center; polymerase chain reaction alone at Moscow City Oncological Hospital No. 62 and SBIH Moscow Clinical Scientific and Practical Center named after A.S. Loginov of DHM; both methods at S.P. Botkin Moscow Multidisciplinary Clinical Center and the National Medical Research Center of Coloproctology. To assess whether diagnostic methodology influenced detection rates, centres were grouped according to their predominant testing approach: immunohistochemistry alone (n=614), polymerase chain reaction-based analysis alone (n=952), or both methods (n=535). MSI/dMMR prevalence did not differ significantly between these groups (9.0%, 11.6%, and 10.3%, respectively; χ² = 2.71; p = 0.257). This centre-level comparison is confounded by between-centre differences in tumour location and stage distribution (see Discussion) and has limited power to detect a modest method-related effect; a patient-level comparison was not possible because testing method was recorded only for MSI/dMMR-positive cases.

## 4 Discussion

To our knowledge, this is the largest prospective multicentre study of mismatch repair deficiency in colorectal cancer conducted in Russia. The prevalence of 10.5% is close to the rates reported in European and North American cohorts (2, 5, 10). This suggests that the frequency of mismatch repair deficiency in colorectal cancer does not differ markedly between Russia and other settings.

The clinicopathological profile of mismatch repair-deficient tumours in our cohort matches the established phenotype of MSI/dMMR colorectal cancer, including proximal location and poor differentiation (2, 13). Right-sided location and poor differentiation were the strongest predictors in both univariate and multivariate models. The association with right-sided tumours was particularly strong and remained robust after multivariable adjustment, consistent with findings from Western cohorts.

The relationship with age turned out to be more complicated than the raw numbers suggested. Patients aged 45 or under had twice the rate of mismatch repair deficiency compared with older patients (20.0% vs. 9.9%), yet mean age was virtually the same across positive and negative groups (66.1 vs. 66.8 years, p = 0.518). In multivariate analysis, older age was an independent inverse predictor. This inverse association is likely explained, at least in part, by the correlation between older age and right-sided tumour location (Spearman rho = 0.144, p < 0.001), which strongly predicts mismatch repair deficiency; given the modest strength of this correlation, confounding by location should be regarded as a plausible contributing mechanism rather than a fully demonstrated explanation. Once location was in the model, the negative association with age became apparent. A formal test for non-linearity using restricted cubic splines provided some additional support for a non-linear age effect (see Results), though this improvement in model fit was modest. As a result, using age alone to select patients for testing would miss many cases — especially in older patients with left-sided or rectal tumours, consistent with prior studies directly comparing universal molecular screening with age- or criteria-based strategies for Lynch syndrome detection (4, 5).

Women accounted for 70.0% of mismatch repair-deficient cases versus 50.1% of proficient cases, and this association persisted after adjustment (odds ratio for male sex 0.49, p < 0.001). A female predominance among MSI/dMMR cases is biologically plausible because most sporadic MSI/dMMR colorectal cancers arise through *MLH1* promoter hypermethylation rather than germline mismatch repair gene mutations (2, 13). However, *BRAF* and *MLH1* methylation data were not available in the present analysis, so the mechanism underlying the observed sex association cannot be determined from this cohort. Notably, both of these indirect markers have recognised limitations for distinguishing sporadic MSI/dMMR from Lynch syndrome: the *BRAF* p.V600E mutation, previously considered a reliable marker of sporadic tumours, has been reported in several cases of Lynch syndrome-associated colorectal cancer (12), and *MLH1* promoter hypermethylation can co-occur with germline pathogenic *MLH1* variants in the same patient (14).

Stage-specific data added further detail. Prevalence peaked at stage II (13.4%; 95% confidence interval 10.7–16.5%) and was lowest at stage IV (6.3%; 95% confidence interval 4.2–8.9%), with stages I and III falling in between (10.2% and 10.9%). The difference across the four stage groups was statistically significant (χ² = 14.056; p = 0.003), and the gap between stage II and stage IV was the largest (odds ratio 0.43; p < 0.001). On multivariate analysis, stage III–IV was independently associated with lower odds of mismatch repair deficiency (odds ratio 0.64), consistent with previous data showing that MSI/dMMR colorectal cancers are relatively under-represented in metastatic disease (11, 13).

None of the clinicopathological predictors — individually or combined — was reliable enough to justify selective testing. A meaningful share of cases fell outside every conventional high-risk category. Mismatch repair status now directly informs eligibility for immune checkpoint inhibitor therapy in advanced colorectal cancer (7–9), so a missed case in the metastatic or locally advanced setting is not just a diagnostic gap — it affects treatment decisions.

The variation in rates across centres (7.7% to 13.8%) was real but explainable. Tumour location distribution differed significantly between institutions (χ² = 67.2, p < 0.001), as did the stage profile (χ² = 91.8, p < 0.001). After accounting for these differences, centre affiliation added nothing to the prediction of mismatch repair status (likelihood ratio test p = 0.133). The rate differences reflect patient mix, not diagnostic practice. Even so, the data point to a need for more standardised testing protocols across Russian oncology centres — something that is directly relevant to current national guideline discussions.

Several limitations apply. Germline testing and survival data were not collected; both will be reported in future registry publications. Testing followed local protocols without central pathology review. Histological grade was missing for 17.8% of patients, mostly at one centre; mismatch repair rates were similar whether grade was recorded or not (10.9% vs. 8.5%, p = 0.176), and adding centre as a covariate did not change the multivariate estimates (p = 0.133). Despite these limitations, the prospective design, consecutive enrolment, and large unselected multicentre cohort address the selection bias that has limited most prior Russian data on this question.

This registry provides the first large-scale real-world estimate of mismatch repair deficiency prevalence across multiple Russian institutions. At 10.5%, the rate matches international data. No clinicopathological profile captures all cases — universal testing is the only approach that does. The centre-to-centre differences we observed point to ongoing standardisation needs. When germline data and survival outcomes become available, the full clinical picture of mismatch repair deficiency in this population will be considerably clearer.

## Data Availability

The raw data supporting the conclusions of this article will be made available by the authors, without undue reservation.

## References

[1] Sung H Ferlay J Siegel RL Laversanne M Soerjomataram I Jemal A . Global cancer statistics 2020: GLOBOCAN estimates of incidence and mortality worldwide for 36 cancers in 185 countries. CA Cancer J Clin. (2021) 71:209–49. doi: 10.3322/caac.21660 33538338

[2] Boland CR Goel A . Microsatellite instability in colorectal cancer. Gastroenterology. (2010) 138:2073–87. doi: 10.1053/j.gastro.2009.12.064 20420947 PMC3037515

[3] Moreira L Balaguer F Lindor N de la Chapelle A Hampel H Aaltonen LA . Identification of Lynch syndrome among patients with colorectal cancer. JAMA. (2012) 308:1555–65. doi: 10.1001/jama.2012.13088 23073952 PMC3873721

[4] Hampel H Frankel WL Martin E Arnold M Khanduja K Kuebler P . Screening for the Lynch syndrome (hereditary nonpolyposis colorectal cancer). N Engl J Med. (2005) 352:1851–60. doi: 10.1056/NEJMoa043146 15872200

[5] Pérez-Carbonell L Ruiz-Ponte C Guarinos C Alenda C Payá A Brea A . Comparison between universal molecular screening for Lynch syndrome and Revised Bethesda Guidelines in a large population-based cohort of patients with colorectal cancer. Gut. (2012) 61:865–72. doi: 10.1136/gutjnl-2011-300041 21868491

[6] Tsukanov AS Pospehova NI Shubin VP Sachkov I Zhdankina SN Ponomarenko AA . Differentiation of Lynch syndrome from other forms of non-polyposis colorectal cancer among Russian patients. Russ J Gastroenterol Hepatol Coloproctol. (2014) 24:78–84.

[7] Le DT Uram JN Wang H Bartlett BR Kemberling H Eyring AD . PD-1 blockade in tumors with mismatch-repair deficiency. N Engl J Med. (2015) 372:2509–20. doi: 10.1056/NEJMoa1500596 26028255 PMC4481136

[8] Rüschoff J Baretton G Bläker H Dietmaier W Dietel M Hartmann A . MSI testing: what’s new? What should be considered? Pathologe. (2021) 42:110–8. doi: 10.1007/s00292-021-00948-3 34477921

[9] Benson AB Venook AP Al-Hawary MM Arain MA Chen YJ Ciombor KK . Colon cancer, version 2.2021. J Natl Compr Canc Netw. (2021) 19:329–59. doi: 10.6004/jnccn.2021.0012 33724754

[10] Kunnackal John G Das Villgran V Caufield-Noll C Giardiello F . Worldwide variation in Lynch syndrome screening: case for universal screening in low colorectal cancer prevalence areas. Fam Cancer. (2021) 20:145–56. doi: 10.1007/s10689-020-00206-0 32914371

[11] Shubin V Shelygin Y Achkasov S Sushkov O Nazarov I Ponomarenko A . Microsatellite instability in Russian patients with colorectal cancer. Int J Mol Sci. (2022) 23:7062. doi: 10.3390/ijms23137062 35806077 PMC9266820

[12] Tsukanov AS Demidova IA Tsaur GA Druy AE Olshanskaya YV Kekeeva TV . Diagnosis of Lynch syndrome in cancer patients: the position of the Interregional Organization of Molecular Geneticists in Oncology and Oncohematology. Voprosy Onkologii. (2023) 69:7–14. doi: 10.37469/0507-3758-2023-69-1-7-14

[13] Sinicrope FA Sargent DJ . Molecular pathways: microsatellite instability in colorectal cancer – prognostic, predictive, and therapeutic implications. Clin Cancer Res. (2012) 18:1506–12. doi: 10.1158/1078-0432.CCR-11-1469 22302899 PMC3306518

[14] Helderman NC Andini KD van Leerdam ME van Hest LP Hoekman DR Ahadova A . MLH1 promotor hypermethylation in colorectal and endometrial carcinomas from patients with Lynch syndrome. J Mol Diagn. (2024) 26:106–14. doi: 10.1016/j.jmoldx.2023.10.005 38061582

